# Texture and Color Enhancement Imaging-Assisted Endocytoscopy Improves Characterization of Gastric Precancerous Conditions: A Set of Interesting Comparative Images

**DOI:** 10.3390/diagnostics15151925

**Published:** 2025-07-31

**Authors:** Riccardo Vasapolli, Johannes Raphael Westphal, Christian Schulz

**Affiliations:** 1 Medical Department II, University Hospital LMU Munich, 81377 Munich, Germany; 2 DZIF Deutsches Zentrum für Infektionsforschung, Partner Site Munich, 81377 Munich, Germany

**Keywords:** endocytoscopy, stomach, preneoplastic lesions, intestinal metaplasia, gastric atrophy, gastric cancer

## Abstract

Chronic atrophic gastritis and intestinal metaplasia (IM) are gastric precancerous conditions (GPCs) associated with an increased risk of gastric cancer. Early detection and accurate characterization of GPC are therefore crucial for risk stratification and the implementation of preventive strategies. In the absence of clear mucosal changes observed through white-light imaging (WLI) or virtual chromoendoscopy, endocytoscopy can help unveil the presence of GPC by enabling in vivo assessment of nuclear and cellular structures at ultra-high magnification. Endocytoscopy is typically performed using WLI following dye-based staining of the mucosa. In this case, we demonstrate that combining endocytoscopy with the texture and color enhancement imaging (TXI) mode substantially improves the assessment of the gastric mucosa. In a 61-year-old man undergoing esophagogastroduodenoscopy, WLI showed multifocal erythema in the stomach, without clearly visible lesions on either WLI or narrow-band imaging. Conventional endocytoscopy revealed multiple small spots of IM with characteristic changes in glandular structures, which were even more evident when using the TXI mode. Histological analysis of targeted biopsies confirmed small foci of IM in both the antrum and corpus. The patient was enrolled in a surveillance program because of his clinical background. The combination of endocytoscopy with the TXI mode significantly enhances the delineation of mucosal and cellular architecture, supporting a more accurate optical diagnosis.

**Figure 1:**
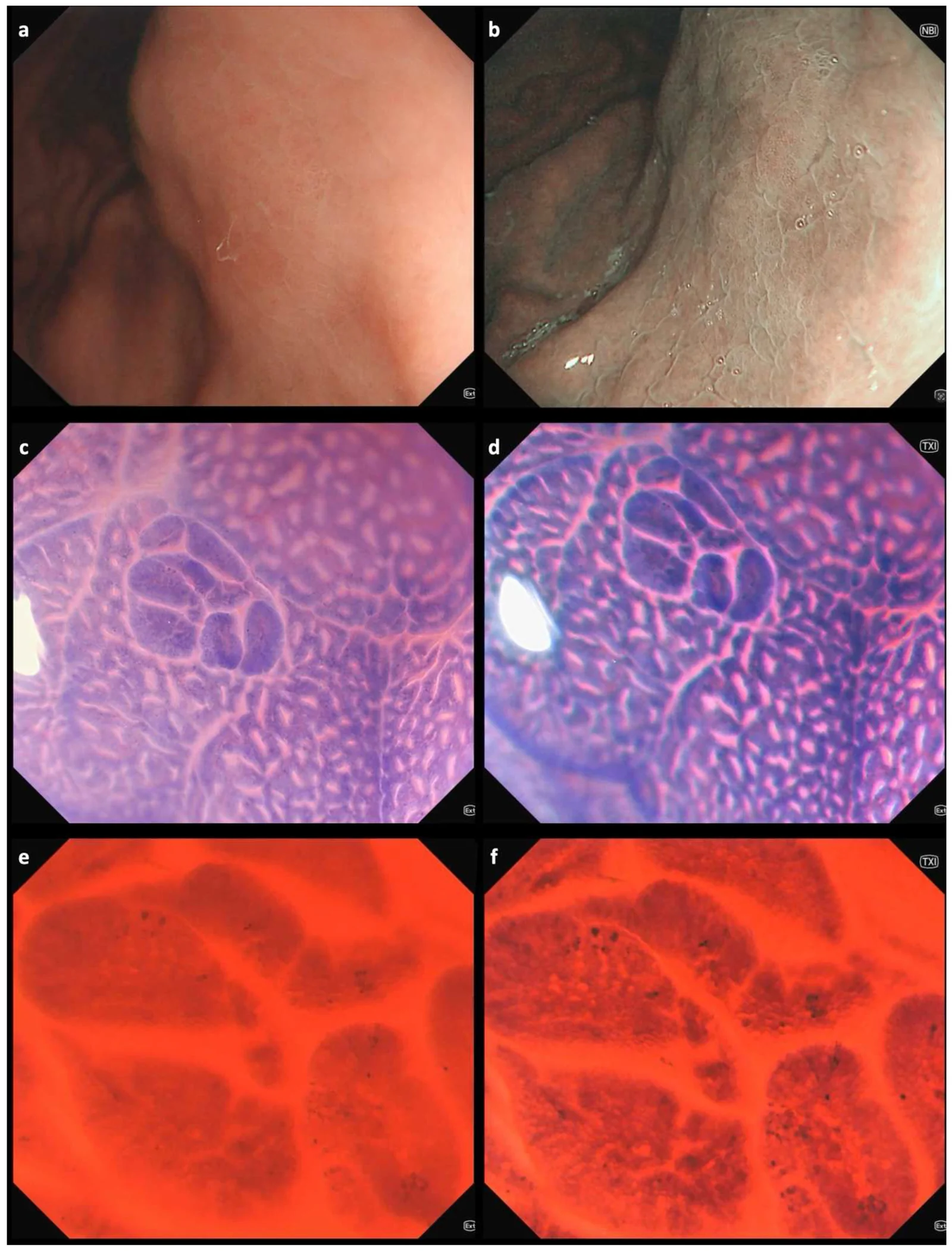
Endoscopic images of a gastric area in the stomach corpus using white-light imaging (WLI) (**a**) and narrow-band imaging (NBI) (**b**), showing mild multifocal erythema and edema. The pit pattern reveals an uneven distribution of gastric pits and the subepithelial capillary network [1,2]; collecting venules are not visible. However, no clearly delineable lesions are observed. Endocytoscopic images from the same gastric area obtained using WLI (**left**) and texture and color enhancement imaging (TXI) (**right**) modes at different magnifications: (**c**,**d**) 150×; (**e**,**f**) 520×. Endocytoscopic assessment of defined lesions is usually performed after double staining with methylene blue and crystal violet [3,4]. In the stomach, repeated staining following mucus removal using mucolytic and defoaming agents may be necessary to obtain clear endocytoscopic images [5]. In our case of a 61-year-old man referred for esophagogastroduodenoscopy due to dyspeptic symptoms, endocytoscopy facilitated the detection of intestinal metaplasia. Characteristic changes observed through endocytoscopy include narrowing of glandular lumens, increased dye uptake, and the presence of goblet cells [6,7]. These features were even more clearly visualized when endocytoscopy was combined with TXI mode (Video S1), which enhances the visualization of structure, color tone, and brightness, thus facilitating the detection of subtle mucosal changes often missed by standard WLI [8]. The patient, who also had a positive family history of gastric cancer, was included in a three-year surveillance program in accordance with current guidelines [9]. Extensive atrophic and metaplastic changes involving both the stomach antrum and corpus are associated with the highest risk of gastric cancer development [10]. These conditions are generally identifiable using modern high-resolution white-light endoscopy combined with image-enhanced modalities such as TXI, NBI, blue laser imaging (BLI), or iScan. TXI is a readily accessible feature on modern Olympus endoscopy systems, activated similarly to NBI by simply pressing a dedicated button, which enhances mucosal texture and color contrast in real time—improving the detection of subtle lesions without requiring additional equipment or significant changes to the endoscopic workflow. Endocytoscopy may serve as a complementary tool in selected cases—particularly when subtle changes remain unclear or when targeted biopsies are required in areas without obvious mucosal alterations, especially in high-risk individuals. The transition from probe-based to fourth-generation endocytoscopy has led to significant technical advancements. In the current system, the endocytoscope is fully integrated into the distal end of a standard gastroscope or colonoscope, eliminating the need for a separate probe [3]. However, despite this progress, several limitations continue to restrict the widespread adoption of the technology. These include the need for dedicated training to obtain high-quality images—typically after a minimum of 30–50 procedures—as well as the requirement for dye-based chromoendoscopy, which can be time-consuming and particularly challenging in the stomach due to mucus interference. Further development is necessary to make endocytoscopy more practical and user-friendly. In this regard, the integration of artificial intelligence is likely to play an important role in enhancing image interpretation and clinical applicability, as suggested by preliminary studies [11,12].

## Data Availability

The original contributions presented in this study are included in the article/Supplementary Materials. Further inquiries can be directed to the corresponding author.

## Supplementary Materials

The following supporting information can be downloaded at: https://www.mdpi.com/article/10.3390/diagnostics15151925/s1, Video S1: Staining and endocytoscopic assessment of an area of intestinal metaplasia in the stomach corpus comparing white-light imaging and texture and color enhancement imaging modes.
